# Automated lung ultrasound image assessment using artificial intelligence to identify fluid overload in dialysis patients

**DOI:** 10.1186/s12882-022-03044-7

**Published:** 2022-12-24

**Authors:** Grace Feng Ling Tan, Tiehua Du, Justin Shuang Liu, Chung Cheen Chai, Chan Maung Nyein, Allen Yan Lun Liu

**Affiliations:** 1 Department of Medicine, Division of Renal Medicine, Khoo Teck Puat Hospital, Singapore, 768828 Singapore; 2 Nanyang Polytechnic, Ave 8, Singapore, 569830 Singapore; 3 Ministry of Health Holdings Pte Ltd, Maritime Square, Singapore, 099253 Singapore

**Keywords:** Haemodialysis, Peritoneal dialysis, Artificial intelligence, Handheld ultrasound, Lung ultrasound, POCUS, Pulmonary oedema, B lines

## Abstract

**Background:**

Fluid assessment is challenging, and fluid overload poses a significant problem among dialysis patients, with pulmonary oedema being the most serious consequence. Our study aims to develop a simple objective fluid assessment strategy using lung ultrasound (LUS) and artificial intelligence (AI) to assess the fluid status of dialysis patients.

**Methods:**

This was a single-centre study of 76 hemodialysis and peritoneal dialysis patients carried out between July 2020 to May 2022. The fluid status of dialysis patients was assessed via a simplified 8-point LUS method using a portable handheld ultrasound device (HHUSD), clinical examination and bioimpedance analysis (BIA).

The primary outcome was the performance of 8-point LUS using a portable HHUSD in diagnosing fluid overload compared to physical examination and BIA. The secondary outcome was to develop and validate a novel AI software program to quantify B-line count and assess the fluid status of dialysis patients.

**Results:**

Our study showed a moderate correlation between LUS B-line count and fluid overload assessed by clinical examination (*r* = 0.475, *p* < 0.001) and BIA (*r* = 0.356. *p* < 0.001). The use of AI to detect B-lines on LUS in our study for dialysis patients was shown to have good agreement with LUS B lines observed by physicians; (*r* = 0.825, *p* < 0.001) for the training dataset and (*r* = 0.844, *p* < 0.001) for the validation dataset.

**Conclusion:**

Our study confirms that 8-point LUS using HHUSD, with AI-based detection of B lines, can provide clinically useful information on the assessment of hydration status and diagnosis of fluid overload for dialysis patients in a user-friendly and time-efficient way.

## Background

Fluid overload is a significant problem among dialysis patients, with increased cardiovascular morbidity and mortality [1]. Many objective methods, e.g. natriuretic peptides, blood volume monitoring, and bioimpedance analysis (BIA), have been explored to guide fluid management in dialysis patients [2, 3]. However, all these methods have limitations and have not shown promising results when used in isolation [4]. Lung ultrasound (LUS) has been shown to estimate lung water reliably, and lung congestion is a risk factor for all-cause and cardiovascular mortality in chronic dialysis patients, even in asymptomatic or mildly symptomatic patients [5, 6]. Most of the time, LUS was performed by trained clinicians or radiographers to reduce the interobserver variability and reliability and increase diagnostic accuracy. Thus, the wider applicability of LUS is usually restricted, and its use is mostly confined to hospital settings. To penetrate the use of LUS as a fluid assessment tool in the community dialysis centre settings, whereby most are staffed by renal nurses and other allied health professionals, we are emergent to develop a more user-friendly and objective fluid assessment strategy by LUS.

Traditionally, the standard 28-site LUS score system developed by Jambrik et al. has been widely adopted to detect interstitial lung water by counting the B lines manually [7]. Studies have shown that a simplified method of LUS using 8 points compared to the traditional 28-point method has a good correlation [8, 9]. It reduces time and complexity to perform and is better suited for everyday clinical practice in dialysis units [9]. To penetrate the use of LUS as a fluid assessment tool in the community dialysis centre settings, whereby most are staffed by renal nurses and other allied health professionals, we are emergent to develop a more user-friendly and objective fluid assessment strategy by LUS. However, a study using HHUSD versus a high-end ultrasound system (HEUS) to assess B-line count in heart failure patients did show fewer B-lines on HHUSD due to the limited clip store capacity of 2-seconds in HHUSD compared to at least 6 seconds in HEUS [10]. We target to standardise the identifications of B lines; regardless of the level of experience of the staff, the measurement method or the complexity of the device, automated detection by artificial intelligence (AI) is demonstrated to be feasible and reliable [11, 12]. Automated B-line detection can be processed by algorithms from deep learning methods [13] and dedicated segmentation, which was shown to be moderately correlated with extracellular lung water [14]. Existing AI software package built-in commercial US systems could also quantify B lines with good agreement from expert review [15]. However, no existing algorithms or software packages have been tested and validated specifically on fluid assessment in dialysis populations.

This study aimed to 1. develop and validate a novel AI software program in dialysis patients on the detection of B-lines by 8-point LUS using a portable HHUSD and 2. evaluate the performance of this program in comparison to physicians’ quantification of B-line count in the fluid assessment of dialysis patients using clinical examination and BIA as references.

## Materials and methods

### Study population and assessments

This was a single-centre study of dialysis patients in Singapore between July 2020 to May 2022. Patients who were reviewed at our hospital’s renal unit with clinical evidence of fluid overload or high interdialytic weight gain were recruited for the study. We included 61 patients (50 haemodialysis [HD], 11 peritoneal dialysis [PD]) in the training phase and 15 patients (10 HD and 5 PD) in the validation phase. All patients were aged 21 years old and above. The fluid status of dialysis patients was assessed using LUS and conventional methods, including clinical examination and BIA. Patients were excluded from the study in the case of (i) pregnancy, (ii) significant co-existent lung disease, (iii) metallic implants (iv) above-ankle or above-wrist amputations. The primary outcome was the performance of 8-point LUS using an HHUSD in diagnosing fluid overload compared to physical examination and BIA. Baseline demographic characteristics, physical examination, vitals and bioimpedance of the participants were recorded. Chest X-ray and echocardiogram results were also recorded if available.

### Clinical examination

Clinical examination of fluid status for HD patients was performed pre- and post-dialysis by the attending physician, while clinical examination was performed once for peritoneal dialysis patients at the time of the study. Clinical examination of fluid status included patient’s vitals, lung examination and peripheral oedema. Based on the clinical examination, patients were classified into euvolemic, mild, moderate or severe fluid overload.

### Lung US and US B-line score

A Philips Lumify portable ultrasound unit equipped with a curved array transducer was used. The investigator was unaware of the patient’s clinical data result when performing the LUS. Two physicians performed LUS for each subject at the same time. All investigators were trained in LUS examination. For HD patients, LUS was performed before and after the dialysis session. For PD patients, a single LUS examination was performed.

8-point LUS consists of bilateral scanning of the chest wall performed with patients in supine or near-to-supine position. The areas were 2 anterior and 2 lateral per side, with each scan consisting of a 2- second ultrasound clip recording (40 frames for each clip). The anterior chest wall was demarcated from the sternum to the anterior axillary line and subdivided into two halves (approximately from the clavicle to the second-third intercostal spaces and from the third space diaphragm). The lateral zone was demarcated from the anterior to the posterior axillary line and was subdivided into upper and basal halves. LUS B lines were recorded in each intercostal space and were defined as a hyperechoic, coherent US bundle at a narrow basis going from the transducer to the limit of the screen [16]. LUS B lines were summed to obtain a score reflecting the extent of lung water accumulation. A total B-line score of 0–4 is normal, while a score of ≥5 indicates lung congestion [17].

### AI image processing software for B line detection

**Fig. 1:**
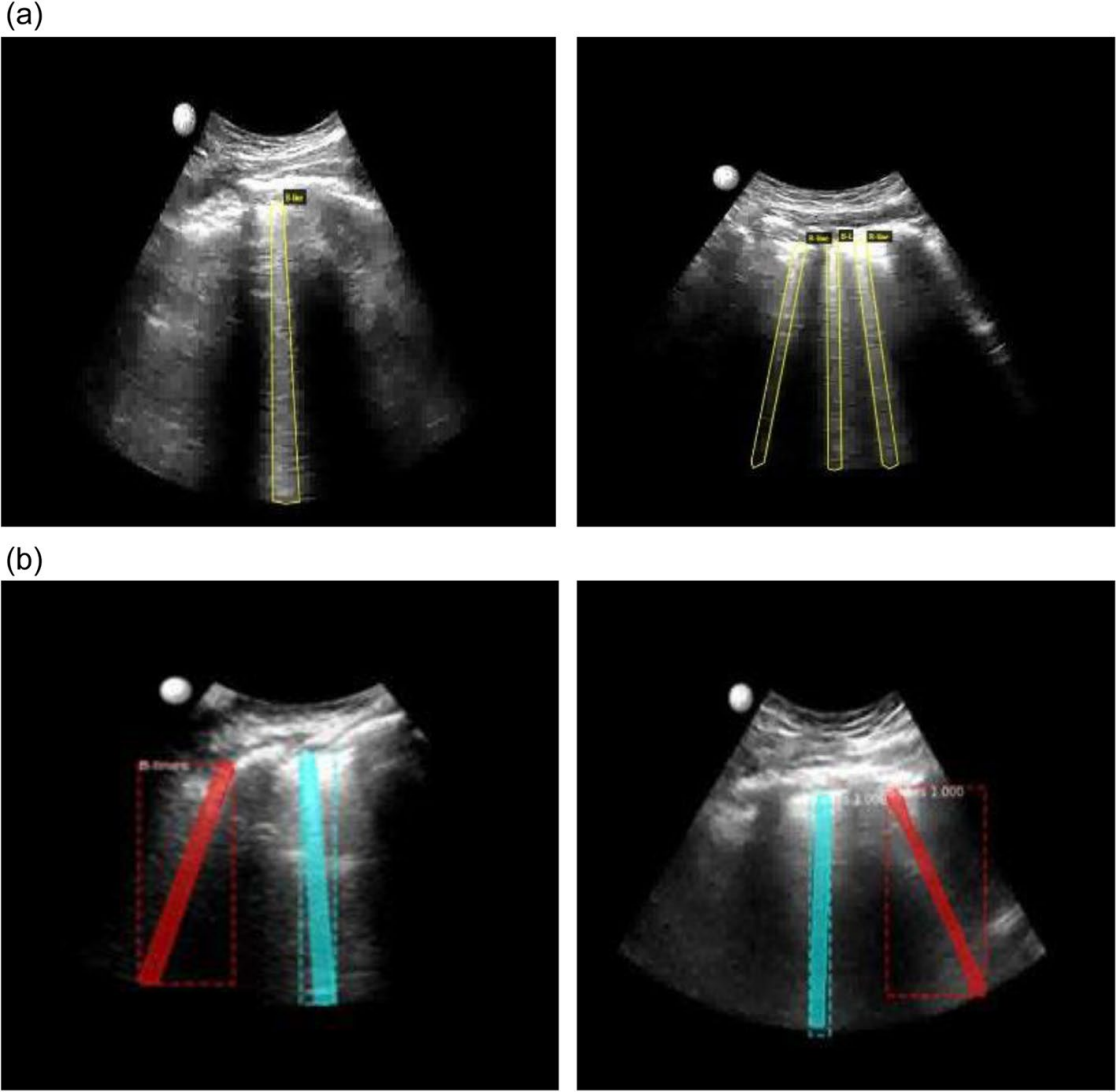
**a** B-lines annotation using VGG and **b** B-lines detected using Mask-RCNN

**Fig. 2:**
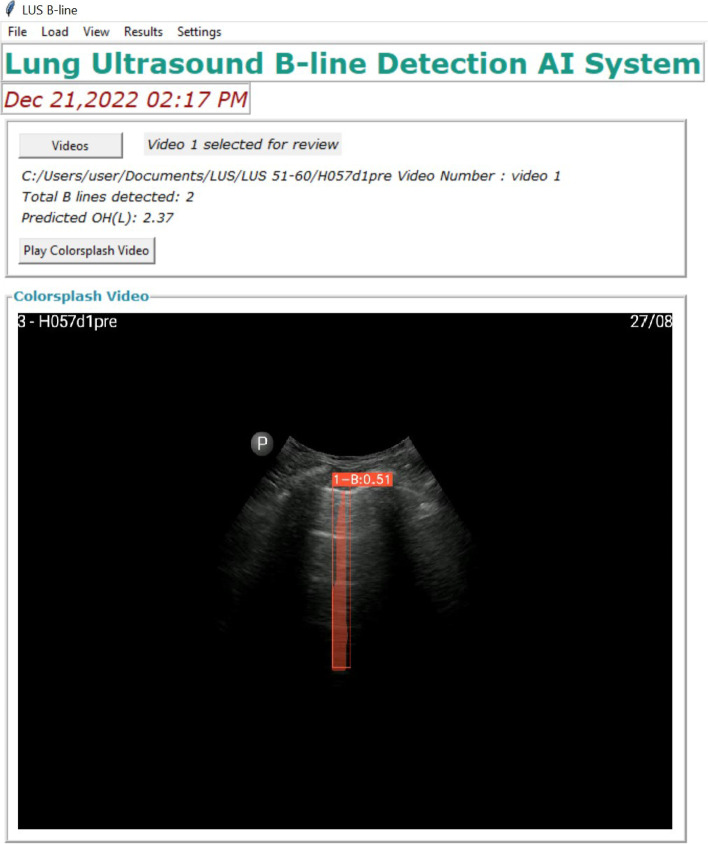
Software program for automated B-line detection

An automated image processing software was developed in collaboration with Nanyang Polytechnic, Singapore. First, LUS videos obtained by the physicians were recorded. A total of 1385 LUS images containing one or more B lines were then labelled, 1003 being used for training and the remaining 382 for validation. VGG image annotator (VIA) was used to trace the outline of each B line that appeared in LUS, as shown in Fig. 1a. We used YOLACT [18] and Mask-RCNN [19] as AI algorithms for instant segmentation. Transfer learning, which could dramatically reduce the number of training images required and training time, was applied to train both models for B-line detection, based on pre-trained weights for Microsoft Common Objects in Context dataset. With a learning rate of 0.001 and minimum detection confidence of 0.9, the Mask-RCNN model converged after 730 iterations. The YOLACT model was also trained using the same labelled data through transfer learning to gain the ability to recognise and segment B lines from other LUS images. The trained model could then segment B lines from a fresh LUS image; an example is shown in Fig. 1b. After that, a B-line tracking algorithm was developed to count the maximum number of B-lines that appeared in each LUS video. A graphic user interface was developed to process LUS videos of a patient and to estimate the fluid overload status (Fig. 2). The software program for automated B-line detection was preinstalled in a commercial laptop computer with a graphic display card for frame analysis. Video frames (eight for each patient) were directly uploaded from the handheld ultrasound device (android interface). The agreement between physicians and AI for B line detection was evaluated and compared.

### Bioimpedance analysis

We used a multi-frequency portable whole-body BIA monitor (Fresenius Medical Care DGmbH). Each HD patient had their body composition measured twice (before and 30 minutes after dialysis). For PD patients, body composition was measured once during the study. BIA has been widely adopted and validated for fluid assessment in dialysis patients [20, 21]. We measured total body water (TBW), extracellular water (ECW), intracellular water (ICW) and volume of distribution of urea (V urea) for each patient. We used overhydration (OH) as a marker of fluid status to compare different measurement strategies, including LUS B-line detection by the physician and AI count, with or without clinically apparent fluid overload.

Ethical approval was obtained from the National Healthcare Group Domain Specific Review Board, Singapore and the study was conducted under the Declaration of Helsinki. Written informed consent was obtained from all participants before enrolment in the study.

### Statistical analysis

Categorical variables were expressed as frequency (percentages) and continuous variables as mean ± standard deviation or median (interquartile range [IQR]) with or without a normal distribution, respectively. The correlation analysis between BIA and LUS measurements was presented as Spearmen’s correlation coefficient. The analysis between physician and AI on LUS B-line count was also expressed as intraclass correlation coefficient (ICC). Using different OH cutoffs, We determined the diagnostic power for physician and AI LUS B-line count as sensitivity, specificity, positive predictive value, negative predictive value and accuracy. Receiver operating characteristics curve (ROC) analysis was performed to evaluate the diagnostic ability of LUS in predicting OH as a dichotomous value. All statistical tests were two-sided, with *p* < 0.05 as statistically significant. All analyses were performed using SPSS 27 (IBM Inc., USA) under licence to Khoo Teck Puat Hospital.

## Results

Baseline clinical characteristics, albumin level, pre-and post-dialysis bedside parameters and pre-dialysis clinical examination on fluid status are shown in Table 1. The inter-rater reliability between physicians was moderately agreed by Cohen’s kappa statistics (0.503, *p* < 0.001). With B lines of ≥5 as cutoff, both groups had comparable age, body mass index, blood pressure, and ejection fraction from an echocardiogram. Patients with B lines ≥5 had more fluid overload from clinical examination (29.5% vs 14.8%). Patients on HD with B lines ≥5 had higher OH extracellular-to-intracellular water ratio (E/I) when measured both pre- and post-dialysis. The differences were not observed in PD patients. Total body water (TBW), extracellular water (ECW) and intracellular water (ICW) were comparable in both HD and PD patients regardless of B line status.

**Table 1:** Baseline clinical characteristics, albumin level, pre- and post-dialysis bedside parameters and pre-dialysis clinical examination on fluid status

	Total (*n* = 61)	B lines ≥5 (*n* = 24)	B lines < 5 (*n* = 37)	*p*-value
Gender (male, %)	35 (57.4)	11 (18.0)	24 (39.3)	0.14
Age (year)	59 ± 11.0	57.6 ± 12.2	60.0 ± 10.2	0.42
Body weight (kg, pre-dialysis)	78.3 ± 17.7	78.2 ± 19.3	78.4 ± 16.8	0.98
Height (m, pre-dialysis)	1.6 ± 0.8	1.6 ± 0.1	1.6 ± 0.1	0.83
SBP (mmHg, pre-dialysis)	148.7 ± 20.9	155.0 ± 19.4	144.6 ± 21.1	0.06
DBP (mmHg, pre-dialysis)	74.1 ± 16.2	74.4 ± 13.5	73.9 ± 17.9	0.9
SpO2 (%, pre-dialysis)	96.9 ± 2.2	96.1 ± 2.0	97.5 ± 2.1	0.01
O2 requirement (L/min, pre-dialysis)	0.8 ± 1.2	1.1 ± 1.4	0.6 ± 1.0	0.09
Albumin (g/dL, pre-dialysis)	32.4 ± 5.0	30.5 ± 5.2	33.7 ± 4.6	0.01
Body weight (kg, post-dialysis)	76.8 ± 17.1	75.4 ± 19.1	77.7 ± 16.0	0.66
SBP (mmHg, postdialysis)	149.8 ± 22.5	154.5 ± 22.8	147.0 ± 22.1	0.25
DBP (mmHg, postdialysis)	73.2 ± 15.3	72.3 ± 14.7	73.8 ± 15.9	0.75
SpO2 (mmHg, postdialysis)	95.6 ± 13.7	97.1 ± 2.4	94.7 ± 17.3	0.56
Echocardiogram EF (%)	49.3 ± 12.1	49.2 ± 12.4	49.5 ± 12.1	0.93
Clinical examination				0.001
Euvolaemic (%)	6 (9.8)	5 (8.2)	1 (1.6)	
Mild fluid overload (%)	28 (45.9)	5 (8.2)	23 (37.7)	
Moderate fluid overload (%)	26 (42.6)	17 (27.9)	9 (14.8)	
Severe fluid overload (%)	1 (1.6)	1 (1.6)	0 (0.0)	
Haemodialysis	**Total (*n*** **= 50)**	**B lines ≥ 5 (*n*** **= 19)**	**B lines < 5 (*n*** **= 31)**	
OH (L, pre-dialysis)	5.2 ± 5.1	7.5 ± 6.6	3.7 ± 3.2	0.01
OH (%, pre-dialysis)	20.6 ± 13.5	27.9 ± 13.7	16.0 ± 11.4	0.003
V urea (L, pre-dialysis)	39.3 ± 12.7	40.9 ± 16.2	38.1 ± 10.1	0.52
BMI (kg/m2, pre-dialysis)	29.7 ± 6.5	29.5 ± 7.3	29.7 ± 6.2	0.92
TBW (L, pre-dialysis)	41.8 ± 12.8	43.7 ± 16.3	40.6 ± 10.3	0.43
ECW (L, pre-dialysis)	21.6 ± 6.8	23.5 ± 8.9	20.5 ± 5.0	0.14
ICW (L, pre-dialysis)	20.3 ± 6.9	20.3 ± 8.2	20.3 ± 6.0	0.99
E/I (pre-dialysis)	1.1 ± 0.2	1.2 ± 0.2	1.0 ± 0.1	0.003
OH (L, post-dialysis)	3.8 ± 5.0	6.0 ± 6.6	2.5 ± 3.1	0.02
OH (%, post-dialysis)	15.4 ± 15.1	22.7 ± 15.2	11.0 ± 13.4	0.007
V urea (L, post-dialysis)	38.8 ± 12.4	39.4 ± 15.8	38.4 ± 10.0	0.77
BMI (kg/m2, post-dialysis)	28.9 ± 6.3	28.6 ± 7.0	29.0 ± 6.0	0.81
TBW (L, post-dialysis)	40.8 ± 12.6	41.9 ± 15.9	40.2 ± 10.4	0.65
ECW (L, post-dialysis)	20.4 ± 6.6	21.7 ± 8.6	19.6 ± 5.0	0.3
ICW (L, post-dialysis)	20.8 ± 7.6	20.8 ± 9.7	20.9 ± 6.1	0.96
E/I (post-dialysis)	1.0 ± 0.2	1.1 ± 0.2	0.9 ± 0.1	0.001
Peritoneal dialysis^*^	**Total (*n*** **= 11)**	**B line ≥ 5 (n = 5)**	**B line < 5 (n = 6)**	
OH (L)	5.0 ± 6.7	8.8 ± 6.1	1.9 ± 5.9	0.09
OH %	19.7 ± 27.6	32.1 ± 15.5	9.4 ± 32.4	0.19
V urea (L)	42.2 ± 12.9	43.7 ± 15.6	40.9 ± 12.5	0.74
BMI (kg/m2)	28.7 ± 6.8	29.8 ± 7.5	27.7 ± 6.8	0.64
TBW (L)	44.7 ± 12.4	46.6 ± 14.7	43.2 ± 11.3	0.67
ECW (L)	22.1 ± 6.2	25.1 ± 8.2	19.6 ± 2.8	0.16
ICW (L)	22.6 ± 8.9	21.5 ± 6.8	23.6 ± 10.8	0.72
E/I	1.0 ± 0.2	1.2 ± 0.1	0.9 ± 0.3	0.12

*All parameters were measured at pre-dialysis for peritoneal dialysis patients

SBP: systolic blood pressure, DBP: diastolic blood pressure, EF: ejection fraction, OH: overhydration, V urea: volume of distribution of urea, BMI: body mass index, TBW, total body water, ECW: extracellular water, ICW: intracellular water, E/I: extracellular/ intracellular water ratio

### LUS and the correlations with clinical examination and BIA

**Fig. 3:**
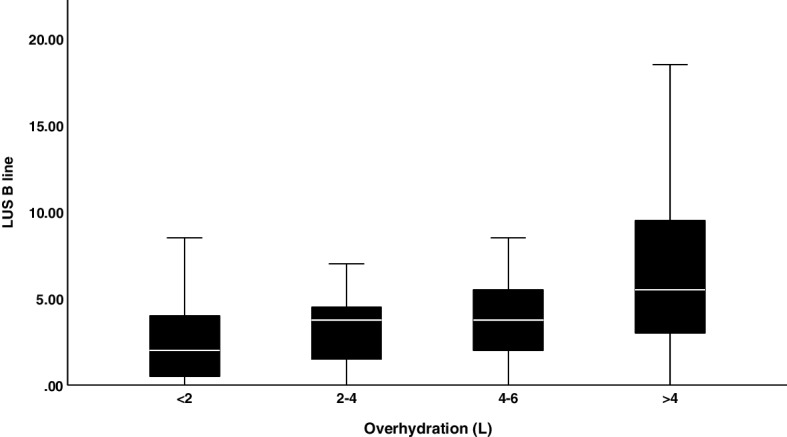
Distribution of LUS B lines by overhydration status

Figure 3 shows the distribution of OH status and the number of B lines. There was a moderate correlation between B lines and OH (*r* = 0.356. *p* < 0.001). LUS B lines were also moderately correlated with clinical examination (*r* = 0.475, *p* < 0.001) and ECW-to-ICW ratio (E/I; *r* = 0.323, *p* = 0.002). It was weakly correlated with the ECW index (ECW divided by body weight; *r* = 0.271, *p* = 0.008). LUS B line was not correlated with age (*r* = − 0.062, *p* = 0.391), body mass index (BMI; *r* = − 0.089, p0.214), LV function by ejection fraction from echocardiogram *r* = − 0.036, *p* = 0.636), TBW (*r* = 0.037, *p* = 0.607), ECW (0.101, *p* = 0.160) and ICW (*r* = − 0.10, *p* = 0.893).

**Fig. 4:**
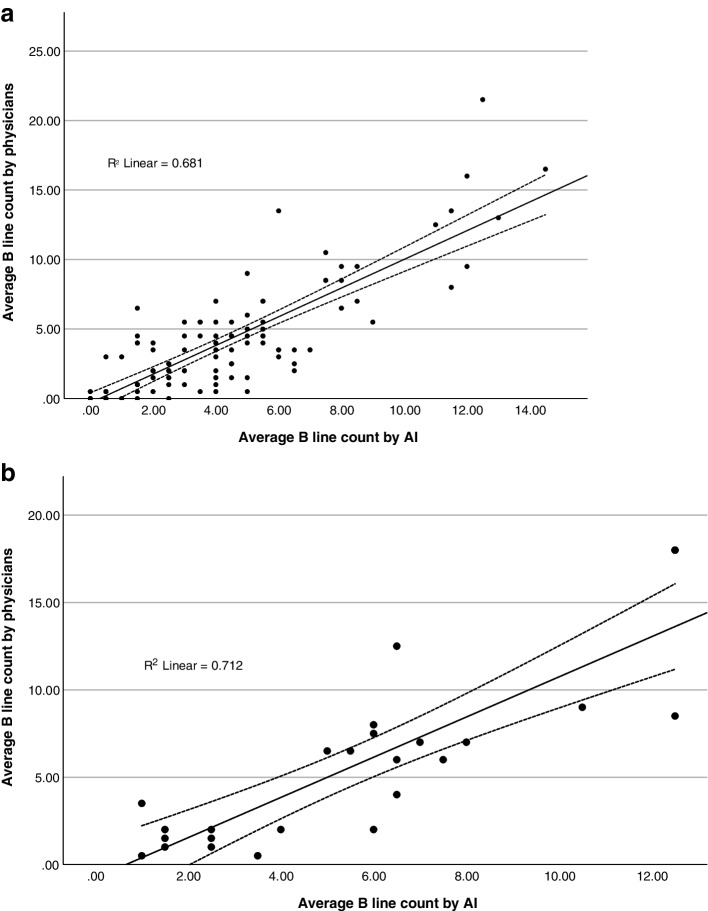
Correlation between B-lines counted by physicians and by AI **a** at training; **b** at validation

Figure 4 shows the scatter plot diagram for the correlation between B lines counted by physicians and by AI. At the training set (Fig. 4a), there was a strong correlation between the two measurements (*r* = 0.825, *p* < 0.001) with an ICC of 0.892 (95% confidence interval [CI] 0.842–0.926, *p* < 0.001). At the validation set (Fig. 4b), The correlation remained strong (*r* = 0.844, *p* < 0.001) with an ICC of 0.892 (95% CI 0.754–0.952, *p* < 0.001).

### Prediction of fluid overload by LUS B lines

**Fig. 5:**
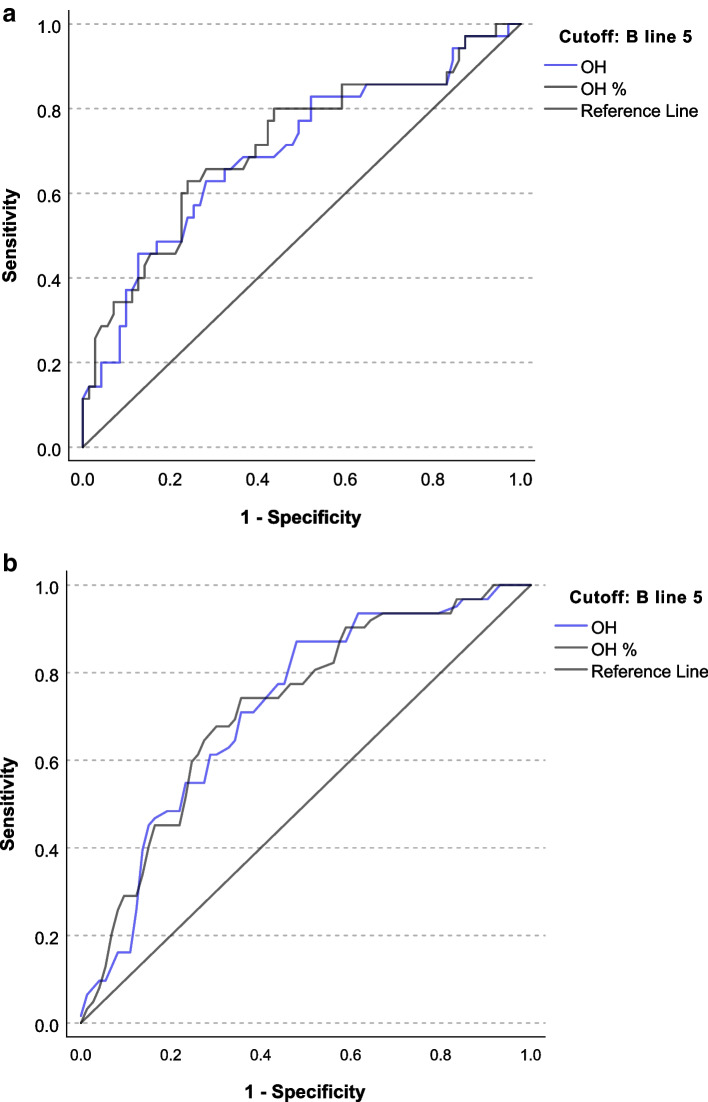
ROC curve for absolute and relative overhydration measured by bioimpedance using B-line ≥5 as cuff-off

**Fig. 6:**
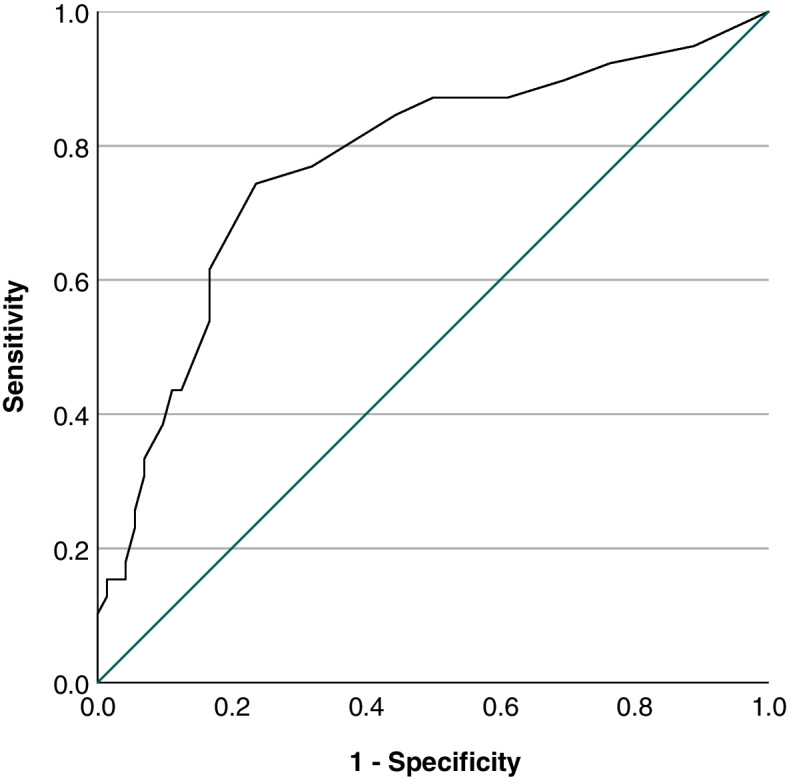
ROC curve for LUS B-line using moderate to severe fluid overload status from clinical examination

The ROC curves of absolute (OH) and relative (OH%) overhydration as a predictor of B lines ≥5 counted by a physician and AI are shown in Fig. 5a and b, respectively. The area under the curve (AUC) was 0.697 (95%CI 0.586–0.808) for OH and 0.713 (95%CI 0.603–0.822) for OH% when physicians counted B lines. AUC was 0.719 (95% CI 0.615–0.82) for OH and 0.721 (95%CI 0.616–0.825) for OH% when AI detected B lines. We also analysed the number of B lines to predict moderate to severe fluid overload from clinical examination. The AUC was 0.773 (95% CI 0.677–0.869, *p* < 0.0001). Figure 6 shows the ROC curve of absolute and relative OH as a predictor of fluid overload by LUS and clinical examination. The optimal number of B lines was 4.5, with sensitivity of 0.744, specificity of 0.764, positive predictive value of 0.667, negative predictive value of 0.853 and accuracy of 0.871.

**Fig. 7:**
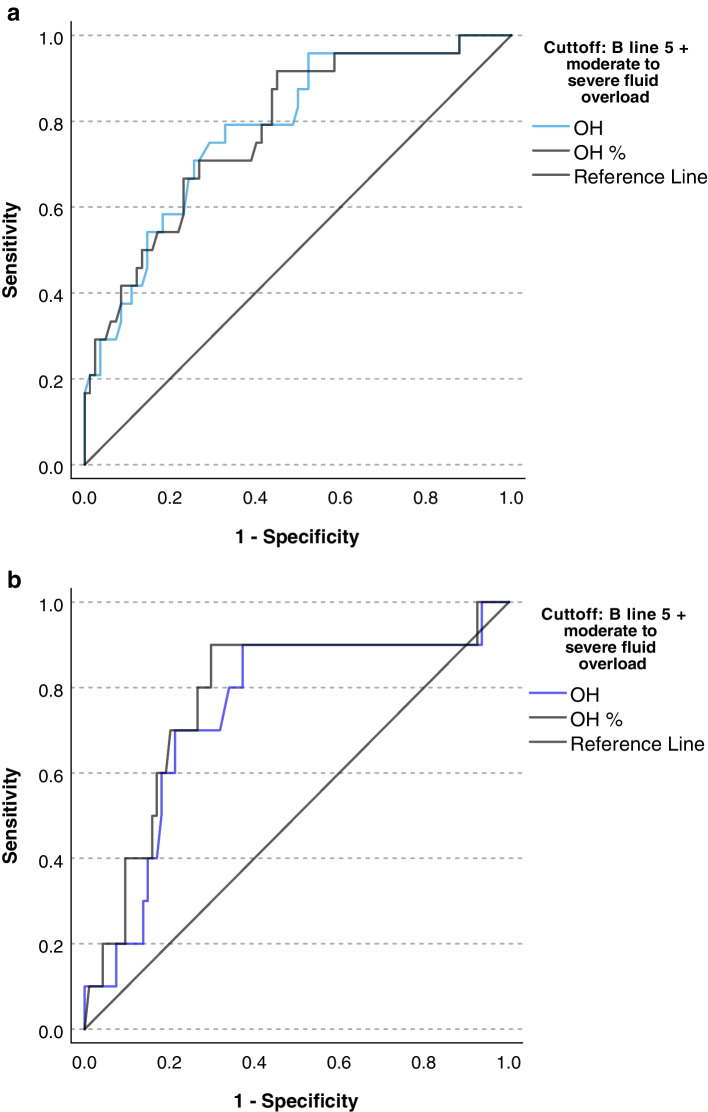
ROC curve for overhydration measured by bioimpedance versus LUS B-line count and clinical examination

If moderate to severe fluid overload status by clinical examination were added together with LUS using B lines ≥5 as a diagnosis of fluid overload, both the AUC for OH (0.782 [95%CI 0.680–0.884]) and OH% (0.781 [0.678–0.883]) increased when physicians counted B lines. The AUC for OH and OH% was 0.743 (95%CI 0.579–0.908) and 0.774 (95% CI 0.611–0.938) when AI was used to detect B lines (Fig. 7).

### Prediction of fluid overload by physician versus AI count of B lines on LUS

**Fig. 8:**
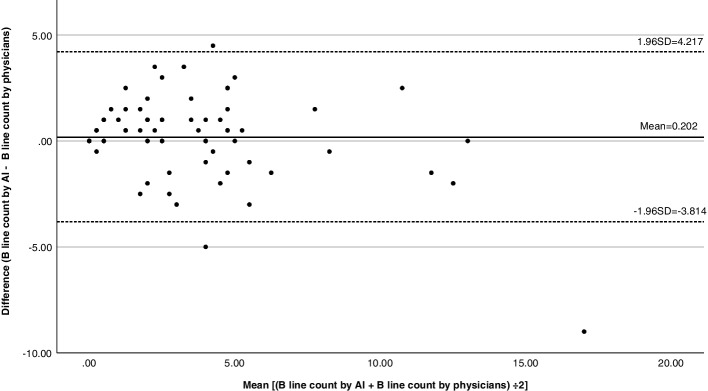
Bland-Altman plot on the agreement between B-line detection from physicians and AI

Table 2 shows the sensitivity, specificity, positive predictive value, negative predictive value and accuracy of different cutoffs for OH for fluid overload defined by B lines ≥5. The accuracy of the two measurements (Physician and AI counts) was similar. Figure 8 shows the Bland-Altman plot on the 95% limits of agreement between B line detection from physicians and AI. The average difference between the two measurements was 0.202. 96% of the samples fell within the limits of agreement.

**Table 2:** Diagnostic ability of fluid overload (LUS B lines ≥5) using different cutoffs of overhydration

		Sensitivity	Specificity	Positive predictive value	Negative predictive value	Accuracy
Physician count	OH > = 4.5	0.639	0.657	0.479	0.787	0.651
AI count	OH > = 4.5	0.636	0.662	0.457	0.803	0.654
Physician count	OH > = 4.75	0.639	0.699	0.511	0.797	0.679
AI count	OH > = 4.75	0.658	0.707	0.511	0.815	0.691
Physician count	OH > = 5	0.583	0.712	0.5	0.776	0.67
AI count	OH > = 5	0.576	0.716	0.475	0.791	0.673
Physician count	OH > =4.5 + clinical	0.76	0.655	0.396	0.902	0.679
AI count	OH > =4.5 + clinical	0.833	0.621	0.217	0.967	0.645
Physician count	OH > =4.75 + clinical	0.76	0.69	0.422	0.906	0.706
AI count	OH > =4.75 + clinical	0.833	0.653	0.233	0.969	0.673
Physician count	OH > =5 + clinical	0.72	0.714	0.429	0.896	0.716
AI count	OH > =5 + clinical	0.75	0.674	0.255	0.955	0.682

## Discussion

### Summary of findings

This study compared the use of 8-point LUS with HHUSD, physical examination, and BIA to diagnose fluid overload. We identified a moderate correlation between the LUS B line and fluid overload by clinical examination and OH by BIA. The prediction of fluid overload by LUS B-line counts was acceptable when using different cutoffs of OH with or without clinical examination as supporting evidence. We found that combined physical examination and LUS to detect fluid overload yielded a better prediction of OH by BIA. This study is the first to validate the use of 8-point LUS to predict OH by BIA in dialysis patients.

Moreover, we used a novel AI LUS B-line detection software program with internal validation by HD and PD patients to evaluate their fluid status. This study showed a strong correlation and good agreement on B line identification between physician and AI count in both training and validation sets. The predictive power of AI LUS B-line detection on the diagnosis of fluid overload was similar to physician count. This study is also the first to use automated LUS assessment to assist in the diagnosis of fluid overload in dialysis patients.

### 8-point LUS in HHUSD

The standard 28-site LUS score system has been extensively applied to HD [5, 22, 23] patients and PD patients [15, 16]. Though learning LUS can be fast and readily available (such as learning from an online video), adopting a short method (like 8-point LUS in our study) can encourage the application of this technique in satellite dialysis centres, where allied health and nursing professionals but not physicians are providing services. 8-point LUS, clinical presentation, and inferior vena cava measurement [24] have recently been applied to HD patients to estimate dry weight. Torino et. el. demonstrated excellent correlation and concordance with 28-point measurement [9]. The same study also showed that 8-point LUS had halved the measurement time compared to the 28-point method.

In our study, we pragmatically combined 8-point measurement with HHUSD to enhance the portability of using LUS. HHUSD has been tested for LUS measurement in HD patients [24, 25]. Miao et al. compared the use of HHUSD with a portable US machine, with comparable and concordant measurements of B lines both before and after HD (*r*^2^ = 0.84), though with a significant bias of 0.06 (actual B line = 1.2, *p* = 0.04). These two types of US machines could be used interchangeably, with HHUSD providing added bandwidth on flexibility, feasibility, and accuracy.

### Clinical examination and LUS on the diagnosis of fluid overload

We demonstrated moderate to severe fluid overload predictability by clinical examination with LUS (AUC = 0.773). In the LUST study [26], clinical examination by auscultation alone in HD patients had a very low discriminatory power for diagnosing moderate and severe lung congestion (AUC = 0.61 and 0.65, respectively). Peripheral oedema was also poorly correlated with the number of B lines in nephrotic syndrome [27], PD [28] and HD [26]. On the other hand, there was a strong relationship between the New York Heart Association (NYHA) class and the number of B lines in both HD [5, 22, 29, 30] and PD [28] patients. While LUS can only detect interstitial lung water, B lines may already be detectable before clinically apparent fluid overload due to increased lung parenchymal permeability [31].

### BIA and LUS on the diagnosis of fluid overload

Our 8-point LUS method yielded a moderate correlation with OH by BIA, similar to other studies [13, 29, 32]. Apart from OH, we also found a significant correlation of LUS B lines with ECW-ICW ratio and ECW index but not TBW or ICW. Siriopol et al. revealed a significant correlation with other fluid-related parameters (TBW, ECW and ICW). Mallamaci et al. denoted a strong association between LUS B lines and left ventricular ejection fraction (*r* = − 0.59, *p* < 0.001), but this was not observed in our study [22]. There are several explanations. First, we included PD patients in this study, for which previous reports revealed conflicting results on the relationship between LUS B lines and hydration status [28, 33]. Second, our study found that the optimal predictive power for detecting fluid overload by LUS was 4.75 L by BIA. This implies that in mild fluid overload (OH < 4 L), the sensitivity and accuracy of detection by LUS will be lowered. In contrast, we recruited some of our HD patients who were hospitalised due to overt fluid overload. Therefore, our results may reflect a higher sensitivity overall than other study populations with less moderate to severe fluid overload status.

### Automated LUS B-line detection in dialysis patients

The use of AI to detect B lines on LUS in our study for dialysis patients was shown to have good agreement with LUS B lines observed by physicians. We also observed similar diagnostic power between physician and AI counts of B lines. Recently various algorithms were built for the measurement of B lines in in vivo [34], intensive care unit [14], emergency department [11] and patients with dyspnoea [15]. The ICC ranged from 0.79–0.94, similar to our findings (0.892, whereas > 0.75 was considered good performance) [11, 12]. Unlike our analysis, none of these studies translated into the diagnostic evaluation of fluid status or correlated to other objective fluid assessments (e.g. BIA). To the best of our knowledge, our study is the first to reveal that automated detection of LUS B lines had similar diagnostic power compared to physicians’ B-line count in different OH cutoffs.

### Clinical applicability

Our study confirms that 8-point LUS using HHUSD, with the automated algorithm-based software interface to assist in the detection of B lines, can provide clinically useful information on the assessment of hydration status and diagnosis of fluid overload for HD and PD patients in a user-friendly and time-efficient way. The information gathered can be particularly useful in decision-making during the prescription of HD and PD in a location where complex and bulky HEUS is not accessible or trained healthcare professionals are not readily available (e.g. satellite dialysis centres). Automated LUS B line detection can be a powerful tool that facilitates nurse-led fluid status assessment for HD and PD patients. Further studies are required to apply this strategy to different clinical outcomes (e.g. intra-dialytic hypotension, hospitalisation and cardiovascular mortality) through external validation and application to different clinical settings.

### Limitations and caveats

Our study has several limitations. First, our results were based on single-centre recruitment of hospitalised HD and PD patients. The generalisability of HHUSD and 8-point LUS to other clinical settings needs further elucidation. However, our validation strongly agreed between physician and AI counts on LUS B lines. Second, there was overall a relatively small number of patients recruited. Third, we did not know the proportion of extracellular lung water in ECW by BIA. Further studies using segmental BIA could provide a more precise relationship between LUS B line measurement and overall body fluid status.

## Conclusion

Our study confirms that 8-point LUS using HHUSD, with an automated algorithm-derived graphic interface to detect B lines, can provide clinically useful information on the hydration status of HD and PD patients. The information gathered can potentially guide decision-making when prescribing HD and PD orders. Further studies are required to validate externally and elucidate the relationship between automated LUS B-line measurement and long-term outcomes in dialysis patients.

## Data Availability

The datasets generated during and/or analysed during the current study are available from the corresponding author on reasonable request.

## Abbreviations

AUC: Area under the curve
AI: Artificial intelligence
BIA: Bioimpedance analysis
HD: Haemodialysis
E/I: Extracellular-to-intracellular
ECW: Extracellular water
HHUSD: Handheld ultrasound device
HEUS: High-end ultrasound system
ICW: Intracellular water
IQR: Interquartile range
ICC: Intraclass correlation coefficient
LUS: Lung ultrasound
NYHA: New York Heart Association
OH: Overhydration
PD: Peritoneal dialysis
ROC: Receiver operating characteristics curve
VIA: Total body waterx
VIA: VGG image annotator
V urea: Volume of distribution of urea
